# Supplementary material for: Antibody-directed evolution reveals a mechanism for enhanced neutralization at the HIV-1 fusion peptide site
Source: Nat Commun. 2023 Nov 21;14:7593. doi: 10.1038/s41467-023-42098-5 (PMC10663459; doi:10.1038/s41467-023-42098-5)
Supplement: Supplementary file 1 — Supplementary Information [file 41467_2023_42098_MOESM1_ESM.pdf]

Antibody Directed Evolution Reveals a Mechanism for Enhanced Neutralization at the HIV-1 Fusion Peptide Site

Supplementary Information

Authors:

Bailey B. Banach[1], Sergei Pletnev[2], Adam S. Olia[2], Kai Xu[2,3], Baoshan Zhang[2], Reda Rawi[2], Tatsiana Bylund[2], Nicole Doria-Rose[2], Thuy Duong Nguyen[4], Ahmed S. Fahad[4], Myungjin Lee[2], Bob C. Lin[2], Tracy Liu[2], Mark Louder[2], Bharat Madan[4], Krisha McKee[2], Sijy O'Dell[2], Mallika Sastry[2], Arne Schön[5], Natalie Bui[4], Chen-Hsiang Shen[2], Jacy R. Wolfe[4], Gwo-Yu Chuang[2], John R. Mascola[2], Peter D. Kwong[2,*], Brandon J. DeKosky[4, 6, 7, 8,*]

Affiliations:

[1] Bioengineering Graduate Program, The University of Kansas, Lawrence, KS 66045, USA

[2] Vaccine Research Center, National Institute of Allergy and Infectious Diseases, National Institutes of Health, Bethesda, MD, 20814, USA

[3] Department of Veterinary Biosciences, The Ohio State University, Columbus, OH 43210, USA

[4] Department of Pharmaceutical Chemistry, The University of Kansas, Lawrence, KS 66045, USA

[5] Department of Biology, John Hopkins University, Baltimore, MD 21218, USA

[6] Department of Chemical Engineering, The University of Kansas, Lawrence, KS 66045, USA.

[7] Department of Chemical Engineering, Massachusetts Institute of Technology, Cambridge, MA 02139, USA.

[8] The Ragon Institute of MGH, MIT, and Harvard, Cambridge, MA 02139, USA

* Corresponding author. Emails: dekosky@mit.edu, pkwong@mail.nih.gov

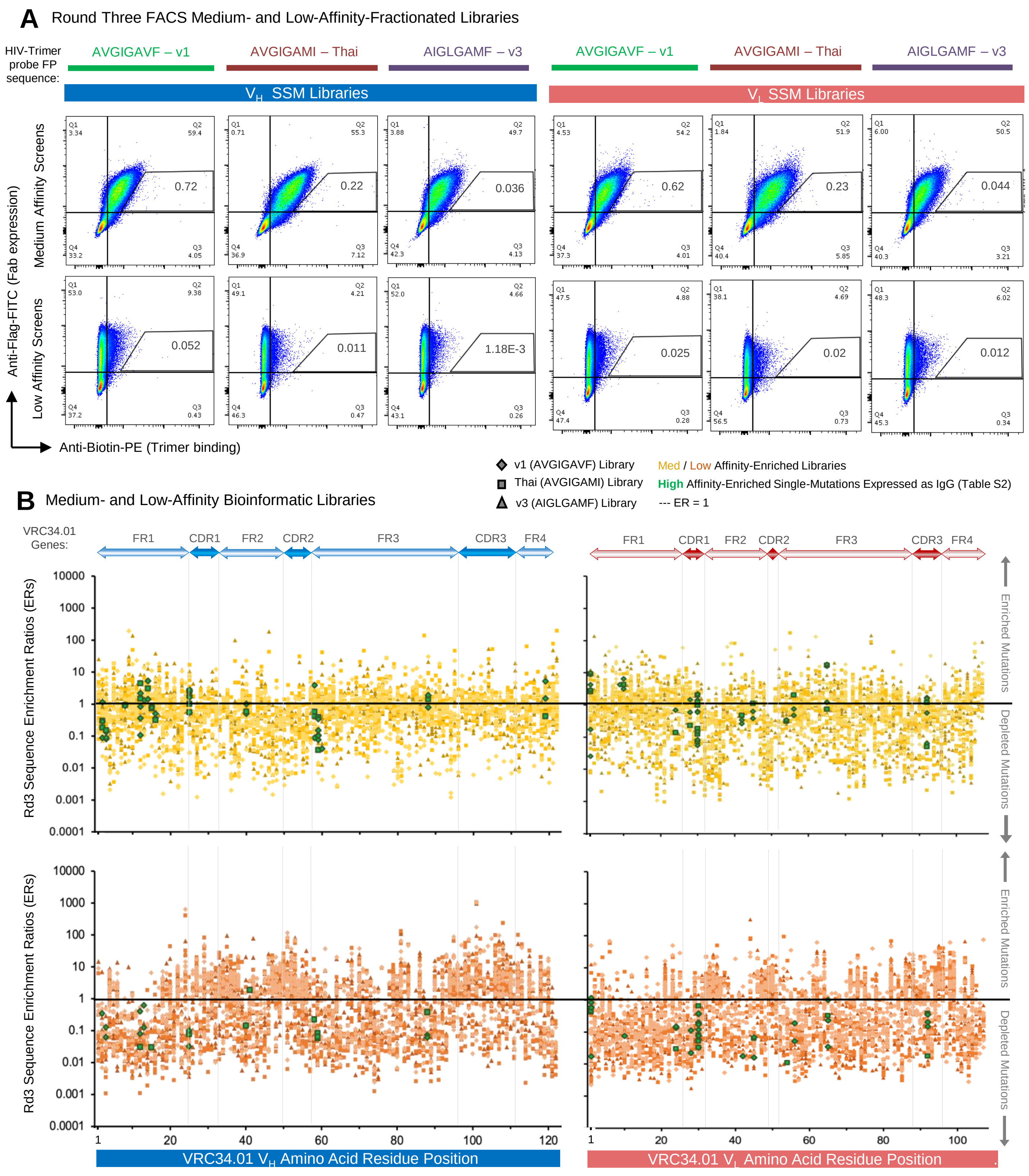

Supplemental Figure 1. FACS and bioinformatic analysis of single mutant library screens used to predict sequence-affinity phenotypes.

A. Medium-affinity (upper), and low-affinity (lower) yeast libraries expressing surface-bound antibodies in a fragment antigen binding (Fab) format, stained with fluorescence markers to measure Fab-surface expression (Y-axis) versus Fab-antigen binding (X-axis) and analyze by flow cytometry.
B. Enrichment ratios (ER) plotted for single mutant antibody sequences derived from Round 3 medium-affinity (top) and low-affinity (bottom) sorted libraries against the mutant residue location in the antibody variable region. Next-generation sequencing (NGS) was used to evaluate library composition across sort rounds to determine individually enriched mutations. NGS data analysis revealed the functional impact of single mutations against diverse FP sequences to select high-affinity candidates for functional and biophysical evaluation.

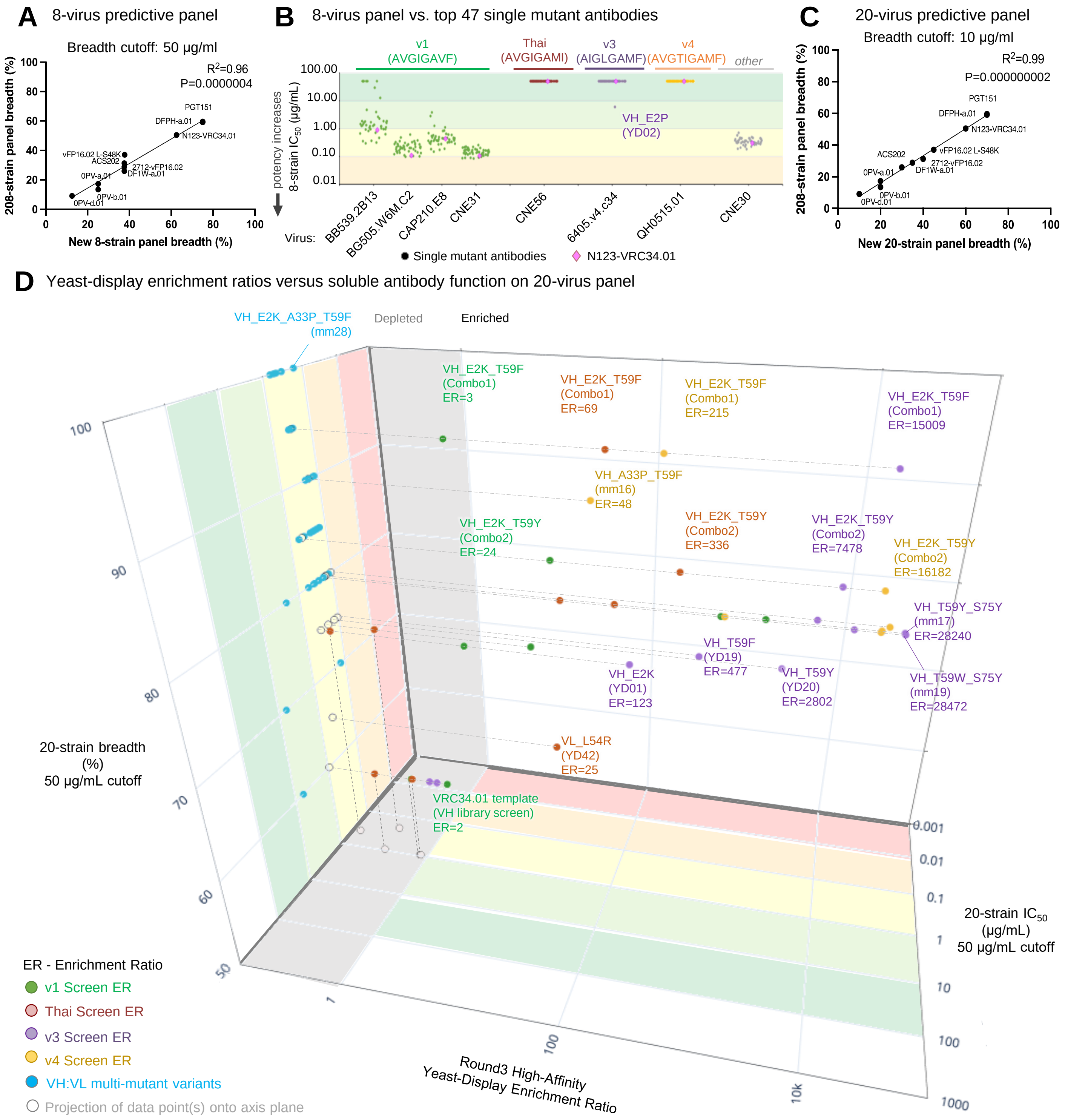

Supplemental Figure 2. Directed antibody evolution informed by predictive neutralization assays.

A. 8-virus predictive neutralization panel design. One-sided p-value for significance of data points deviating from a straight line is shown. No adjustments were made for multiple comparisons.
B. Top 47 enriched single mutant IgG neutralization function on the predictive 8 virus panel.
C. 20-virus predictive neutralization panel design. One-sided p-value for significance of data points deviating from a straight line is shown. No adjustments were made for multiple comparisons.
D. Yeast-display enrichment vs. soluble antibody function on the 20-virus panel shows that higher yeast-display screening enrichment ratios were correlated with improved antibody function.

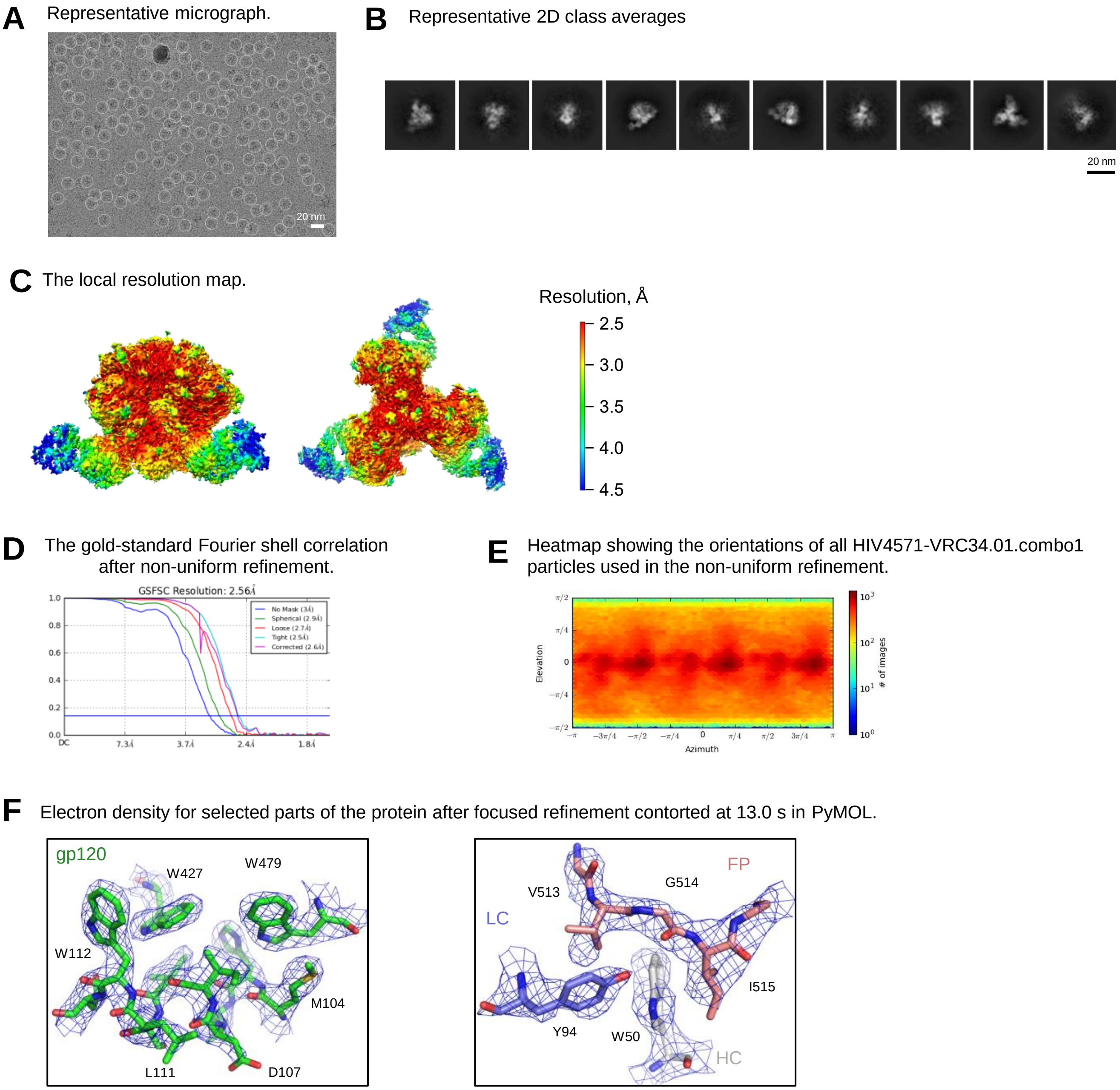

Supplemental Figure 3. Cryo-EM validation of the HIV4571-VRC34.01.combo1 complex.

A. Representative micrograph. 8,020 micrographs were collected. The scale bar is 20 nm.
B. Representative 2D class averages.
C. The local resolution map.
D. The gold-standard Fourier shell correlation after non-uniform refinement.
E. Heatmap showing the orientations of all HIV4571-VRC34.01.combo1 particles used in the non-uniform refinement.
F. Electron density for selected parts of the protein after focused refinement contorted at 13.0 s in PyMOL.

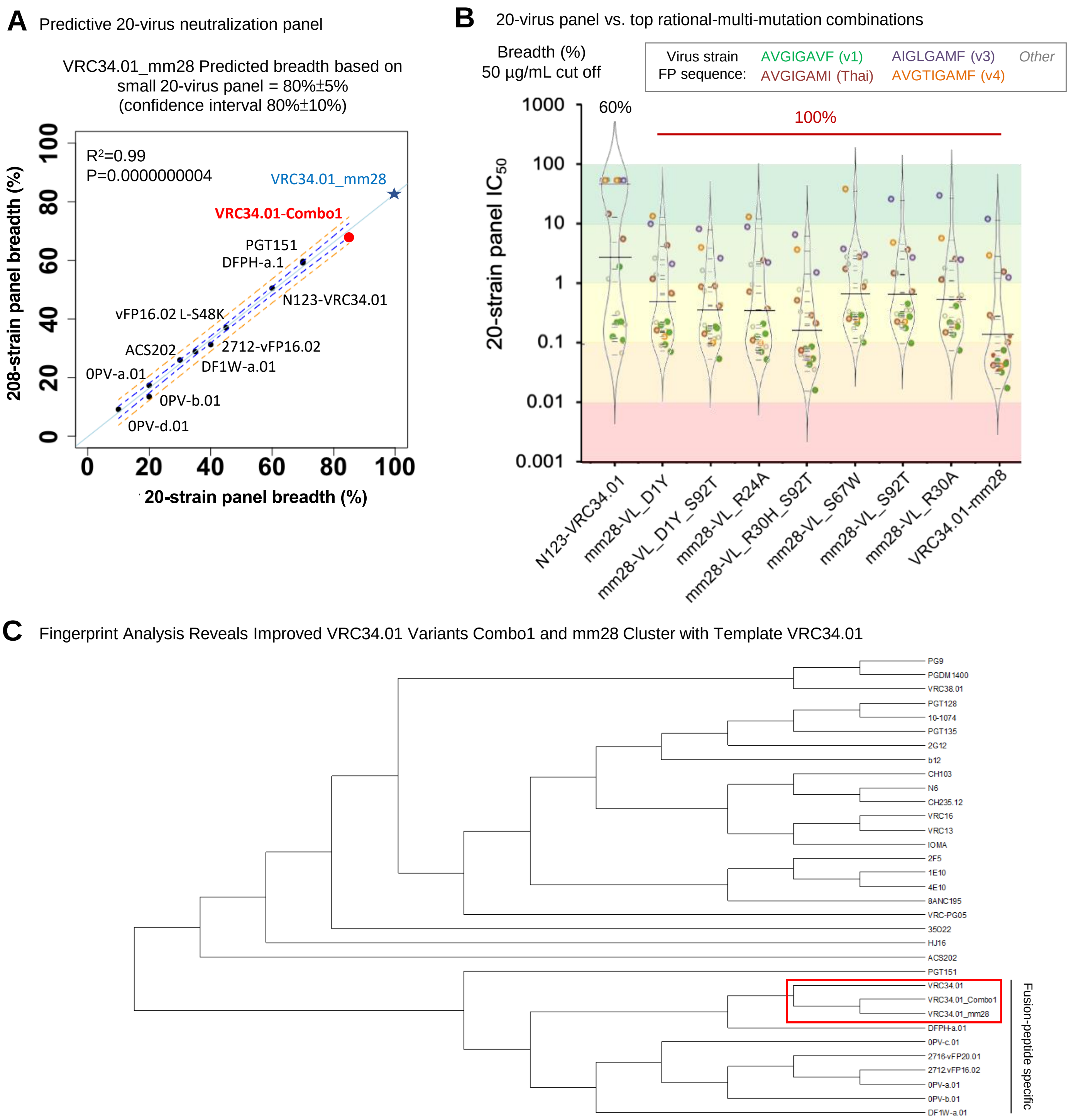

Supplemental Figure 4. Top VRC34.01 mutant variant characterization.

A. VRC34.01_mm28 predicted breadth on a 208-virus panel, based on 20-virus panel predictive panel (80%+/-5% confidence interval 80%+/-10%). Because no reported anti-FP antibody has been reported with as high breadth as VRC34.01_mm28, this prediction required extrapolation beyond the available data points. One-sided p-value for significance of data points deviating from a straight line is shown.
B. Multi-mutation 20-virus panel results, including mm28.
C. VRC34.01_Combo1 neutralization fingerprint analysis indicated that the VRC34.01 template and engineered variants neutralized similar patterns of HIV-1 strains.

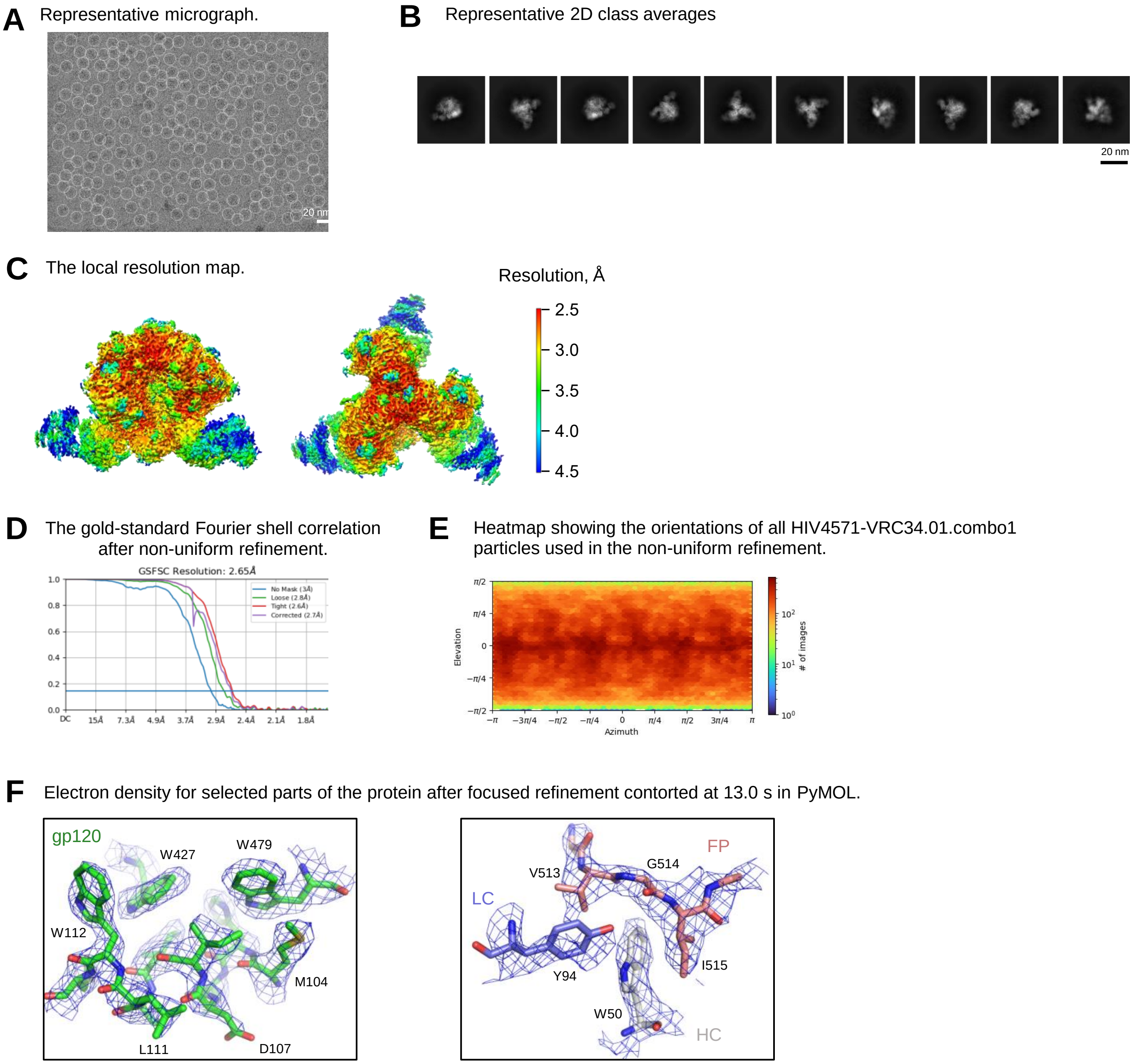

Supplemental Figure 5. Cryo-EM validation of HIV4571-VRC34.01 complex.

A. Representative micrograph. 8,085 micrographs were collected. The scale bar is 20 nm.
B. Representative 2D class averages.
C. The local resolution map.
D. The gold-standard Fourier shell correlation after non-uniform refinement.
E. Heatmap showing the orientations of all HIV4571-VRC34.01 particles used in the non-uniform refinement.
F. Electron density for selected parts of the protein after focused refinement contorted at 13.0 s in PyMOL.

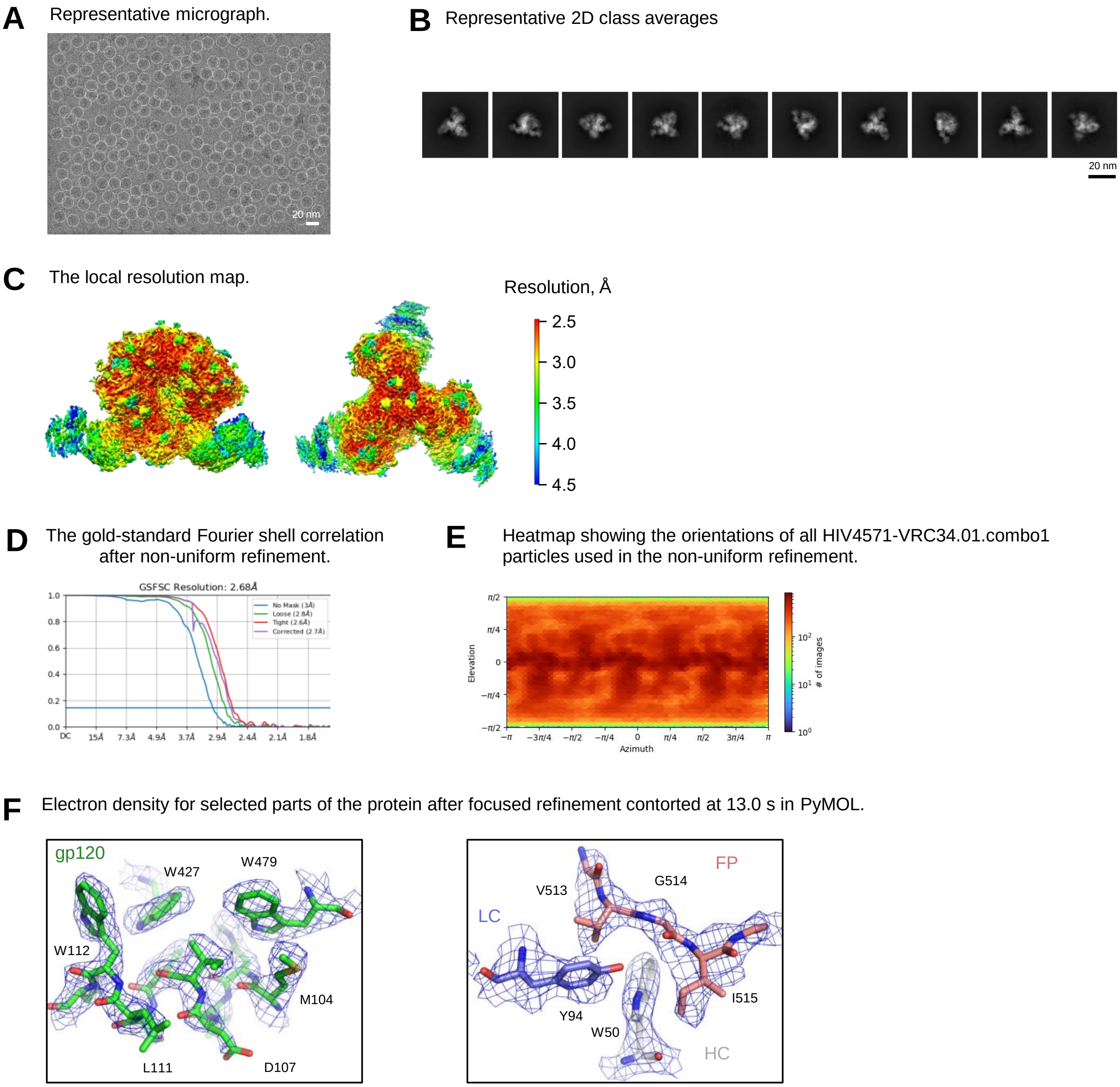

Supplemental Figure 6. Cryo-EM validation of HIV4571-VRC34.01.mm28 complex.

A. Representative micrograph. 9,616 micrographs were collected. The scale bar is 20 nm.
B. Representative 2D class averages.
C. The local resolution map.
D. The gold-standard Fourier shell correlation after non-uniform refinement.
E. Heatmap showing the orientations of all HIV4571-VRC34.01.mm28 particles used in the non-uniform refinement.
F. Electron density for selected parts of the protein after focused refinement contorted at 13.0 s in PyMOL.

Supplementary Tables

Supplementary Table 1. VRC34.01 mutant-library generation & cloning statistics.

		Single Mutant Libraries		Multi-Mutant Libraries				
	Library Name:	VRC34.01-VH-SSM	VRC34.01-VL-SSM	Library 1	Library 2	Library 3	Library 4	Library 5
	Gene augmentation strategy	NNK codon substitution	MNN codon substitution	Rd3-High VH:VL	Rd3-High-VH-SSM : Template-VL	Template-VH : Rd3-High-VL-SSM	Rd3-High-Shuffled VH:VL	Rd3-High-Shuffled-SSM VH:VL
	*Theoretical Number of Variants	3,904	3,424	*unknown*	*unknown*	*unknown*	*unknown*	*unknown*
E. coli transformations	# Transformants	4.00E+06	3.70E+06	1.80E+07	4.50E+07	4.30E+07	Transf.1 (Shuff-VH) - 9.90E+07, Transf.2 (Shuff-VL) - 8.40E+07	Transf.1 (VH-SSM) - 1.80E+8, Transf.2 (VL-SSM) - 1.55E+08
	Transformed clones with single mutations	7 out of 12	10 out of 12	*N/A*	*N/A*	*N/A*	*N/A*	*N/A*
	Adujsted Fold Coverage Estimate	598	900	*N/A*	*N/A*	*N/A*	*N/A*	*N/A*
Yeast transformations	# Transformants	2.10E+06	2.90E+06	4.50E+06	4.90E+06	5.90E+06	6.90E+06	3.20E+06
	Theoretical Fold Coverage	538	847	*unknown*	*unknown*	*unknown*	*unknown*	*unknown*

*Theoretical number of variants calculated as (Length of variable gene) * (No. of possible codons)
VRC34.01-VH-SSM: 122 residues * 32 codons = 3,904 possible single mutant variants
VRC34.01-VL-SSM: 107 residues * 32 codons = 3,424 possible single mutant variants

Supplementary Table 2. VRC34.01 Variants Expressed as IgG. Mutations are written as *original_AA-number-substituted_AA*. Mutations are shown in template protein numbering, as well as Kabat numbering.

Single-mutant variants identified by yeast-library screening

	Antibody IgG	Heavy chain	Heavy chain Kabat ID	Light chain
1	YD01	VRC34.01-HC-E2K	E2K	VRC34.01 L
2	YD02	VRC34.01-HC-E2P	E2P	VRC34.01 L
3	YD03	VRC34.01-HC-V3W	V3W	VRC34.01 L
4	YD04	VRC34.01-HC-V3F	V3F	VRC34.01 L
5	YD05	VRC34.01-HC-G8D	G8D	VRC34.01 L
6	YD06	VRC34.01-HC-K12A	K12A	VRC34.01 L
7	YD07	VRC34.01-HC-K12T	K12T	VRC34.01 L
8	YD08	VRC34.01-HC-K13L	K13L	VRC34.01 L
9	YD09	VRC34.01-HC-P14Q	P14Q	VRC34.01 L
10	YD10	VRC34.01-HC-G15E	G15E	VRC34.01 L
11	YD11	VRC34.01-HC-A16D	A16D	VRC34.01 L
12	YD12	VRC34.01-HC-F25A	F25A	VRC34.01 L
13	YD13	VRC34.01-HC-F25G	F25G	VRC34.01 L
14	YD14	VRC34.01-HC-F25L	F25L	VRC34.01 L
15	YD15	VRC34.01-HC-A40T	A40T	VRC34.01 L
16	YD16	VRC34.01-HC-P41Y	P41Y	VRC34.01 L
17	YD17	VRC34.01-HC-T58Q	T57Q	VRC34.01 L
18	YD18	VRC34.01-HC-T58V	T57V	VRC34.01 L
19	YD19	VRC34.01-HC-T59F	T58F	VRC34.01 L
20	YD20	VRC34.01-HC-T59Y	T58Y	VRC34.01 L
21	YD21	VRC34.01-HC-T59W	T58W	VRC34.01 L
22	YD22	VRC34.01-HC-T59V	T58V	VRC34.01 L
23	YD23	VRC34.01-HC-T60L	T59L	VRC34.01 L
24	YD24	VRC34.01-HC-T60V	T59V	VRC34.01 L
25	YD25	VRC34.01-HC-S88G	S84G	VRC34.01 L
26	YD26	VRC34.01-HC-T119M	T115M	VRC34.01 L
27	YD27	VRC34.01 H	*Wild Type*	VRC34.01-LC-D1G
28	YD28	VRC34.01 H	*Wild Type*	VRC34.01-LC-D1I
29	YD29	VRC34.01 H	*Wild Type*	VRC34.01-LC-D1Y
30	YD30	VRC34.01 H	*Wild Type*	VRC34.01-LC-S9D
31	YD31	VRC34.01 H	*Wild Type*	VRC34.01-LC-F10I
32	YD32	VRC34.01 H	*Wild Type*	VRC34.01-LC-R24D
33	YD33	VRC34.01 H	*Wild Type*	VRC34.01-LC-G28H
34	YD34	VRC34.01 H	*Wild Type*	VRC34.01-LC-R30A
35	YD35	VRC34.01 H	*Wild Type*	VRC34.01-LC-R30F
36	YD36	VRC34.01 H	*Wild Type*	VRC34.01-LC-R30Q
37	YD37	VRC34.01 H	*Wild Type*	VRC34.01-LC-R30S
38	YD38	VRC34.01 H	*Wild Type*	VRC34.01-LC-R30W
39	YD39	VRC34.01 H	*Wild Type*	VRC34.01-LC-R30Y
40	YD40	VRC34.01 H	*Wild Type*	VRC34.01-LC-K42R
41	YD41	VRC34.01 H	*Wild Type*	VRC34.01-LC-N45T
42	YD42	VRC34.01 H	*Wild Type*	VRC34.01-LC-L54R
43	YD43	VRC34.01 H	*Wild Type*	VRC34.01-LC-S56H
44	YD44	VRC34.01 H	*Wild Type*	VRC34.01-LC-T65R
45	YD45	VRC34.01 H	*Wild Type*	VRC34.01-LC-T65S
46	YD46	VRC34.01 H	*Wild Type*	VRC34.01-LC-S92F
47	YD47	VRC34.01 H	*Wild Type*	VRC34.01-LC-S92T

Rational combinations of top single mutants identified by yeast-library screening

	Antibody IgG	Heavy chain	Light chain
1	VRC34.01_Combo1	VH_E2K_T59F	VRC34.01 L
2	VRC34.01_Combo2	VH_E2K_T59Y	VRC34.01 L
3	*VRC34.01_Combo3	VH_E2P_T59F	VRC34.01 L
4	*VRC34.01_Combo4	VH_E2P_T59Y	VRC34.01 L

*Did not express

Multi-mutant (mm) variants identified by yeast-library screening

	Antibody IgG	Heavy chain	Light Chain
1	VRC34.01_mm1	VH_E2K_T59F	VL_D1Y
2	VRC34.01_mm2	VH_E2K_T59F	VL_D1Y_S92T
3	VRC34.01_mm3	VH_E2K_T59F	VL_R24A
4	VRC34.01_mm4	VH_E2K_T59F	VL_R30H_S92T
5	VRC34.01_mm5	VH_E2K_T59F	VL_S67W
6	VRC34.01_mm6	VH_E2K_T59F	VL_S92T
7	VRC34.01_mm7	VH_ E2K_K12A_T59F	VL_D1Y
8	VRC34.01_mm8	VH_E2K_K12A_T59F	VL_D1Y_S92T
9	VRC34.01_mm9	VH_E2K_K12T_T59F	VL_D1Y
10	VRC34.01_mm10	VH_E2K_K12T_T59F	VL_D1Y_S92T
11	VRC34.01_mm11	VH_K12A_T59F_S75Y	VL_R24A
12	VRC34.01_mm12	VH_T59F	VL_S67W
13	VRC34.01_mm13	VH_T59F	VL_D1Y_S92T
14	VRC34.01_mm14	VH_T59F	VL_R30H_S92T
15	VRC34.01_mm15	VH_T59F	VL_R30A
16	VRC34.01_mm16	VH_A33P_T59F	VRC34.01 L
17	VRC34.01_mm17	VH_T59Y_S75Y	VRC34.01 L
18	VRC34.01_mm18	VH_T59Y_S75W	VRC34.01 L
19	VRC34.01_mm19	VH_T59W_S75Y	VRC34.01 L
20	VRC34.01_mm20	VH_T59F_S75W	VRC34.01 L
21	VRC34.01_mm21	VH_T59F_S75Y	VL_R24A
22	VRC34.01_mm22	VH_E2K_T59F_S75Y	VRC34.01 L
23	VRC34.01_mm23	VH_E2K_T28N_T59F	VRC34.01 L
24	VRC34.01_mm24	VH_E2K_T59F_S75Y	VL_R30H_S92T
25	VRC34.01_mm25	VH_E2K_T59F_S75Y	VL_S67W
26	VRC34.01_mm26	VH_E2K_T59F_S75Y	VL_S92T
27	VRC34.01_mm27	VH_E2K_T59F_S75W	VRC34.01 L
28	VRC34.01_mm28	VH_E2K_A33P_T59F	VRC34.01 L

Rational combinations of top multi-mutants identified by yeast-library screening

	Antibody IgG	Heavy chain	Light chain
1	mm28_VL_D1Y	VH_E2K_A33P_T59F	VL_D1Y
2	mm28_VL_D1Y_S92T	VH_E2K_A33P_T59F	VL_D1Y_S92T
3	mm28_VL_R24A	VH_E2K_A33P_T59F	VL_R24A
4	mm28_VL_R30H_S92T	VH_E2K_A33P_T59F	VL_R30H_S92T
5	mm28_VL_S67W	VH_E2K_A33P_T59F	VL_S67W
6	mm28_VL_S92T	VH_E2K_A33P_T59F	VL_S92T
7	mm28_VL_R30A	VH_E2K_A33P_T59F	VL_R30A

Supplementary Table 3. 8-virus predictive neutralization panel design, related to Figure S2B.

			Control Antibodies IC50 (ug/mL)								
**Strains**	**Clade**	**FP seq**	**ACS202**	**N123-VRC34.01**	**PGT151**	**DF1W314**	**0PV12**	**0PV20**	**0PV21**	**110D12**	**2712-vFP16.02**
BG505.W6M.C2	A	**AVGIGAVF – v1**	50	0.211	0.004	0.257	15.2	6.07	20.7	2.21	2.51
CNE30	C	**AVGLGAVF – other**	50	0.191	50	500	500	500	500	5.29	100
BB539.2B13	A	**AVGIGAVF – v1**	50	1.82	0.005	0.566	460	500	122	10.9	100
CAP210.E8	C	**AVGIGAVF – v1**	1.02	0.218	0.032	500	500	101	500	2.66	43.8
CNE31	C	**AVGIGAVF – v1**	0.037	0.122	0.01	314	500	500	500	5.2	100
CNE56	AE	**AVGIGAMI – Thai**	50	500	50	500	3.19	24.6	133	0.65	0.405
6405.v4.c34	D	**AIGLGAMF – v3**	50	500	0.036	28.9	500	500	500	50	100
QH0515.01	B	**AVGTIGAMF – v4**	0.422	500	0.009	500	500	500	500	50	100

Supplementary Table 4. 8-virus predictive neutralization panel data for 47 VRC34.01 single mutation variants, related to Figure S2B.

IC50

			clade	A	A	AE	B	C	C	C	D
source	Descriptive ID	Antibody	virus	BB539.2B13.SG3	BG505.W6M.C2.SG3	CNE56.SG3	QH0515.01.SG3	CAP210.E8.SG3	CNE30.SG3	CNE31.SG3	6405.v4.c34.SG3
BZ	VRC34.01-HC-E2K	VRC34.01-YD01		0.3000	0.1360	50.0000	50.0000	0.1510	0.2090	0.1180	50.0000
BZ	**VRC34.01-HC-E2P**	VRC34.01-YD02		0.3150	0.1030	50.0000	50.0000	0.1890	0.2300	0.0930	6.0600
BZ	VRC34.01-HC-V3W	VRC34.01-YD03		50.0000	0.1530	50.0000	50.0000	2.8300	0.5740	0.2170	50.0000
BZ	VRC34.01-HC-V3F	VRC34.01-YD04		50.0000	0.1890	50.0000	50.0000	1.2500	0.4460	0.1970	50.0000
BZ	VRC34.01-HC-G8D	VRC34.01-YD05		1.8800	0.1840	50.0000	50.0000	0.3170	0.3510	0.1660	50.0000
BZ	VRC34.01-HC-K12A	VRC34.01-YD06		1.6800	0.1990	50.0000	50.0000	0.4180	0.4290	0.1710	50.0000
BZ	VRC34.01-HC-K12T	VRC34.01-YD07		1.8200	0.1620	50.0000	50.0000	0.5840	0.4670	0.1780	50.0000
BZ	VRC34.01-HC-K13L	VRC34.01-YD08		1.8500	0.1320	50.0000	50.0000	0.2900	0.3760	0.1480	50.0000
BZ	VRC34.01-HC-P14Q	VRC34.01-YD09		1.4500	0.1370	50.0000	50.0000	0.3580	0.3600	0.1200	50.0000
BZ	VRC34.01-HC-G15E	VRC34.01-YD10		1.3800	0.2700	50.0000	50.0000	0.4480	0.4500	0.1350	50.0000
BZ	VRC34.01-HC-A16D	VRC34.01-YD11		1.4600	0.2820	50.0000	50.0000	0.6940	0.4180	0.2070	50.0000
BZ	VRC34.01-HC-F25A	VRC34.01-YD12		50.0000	0.2750	50.0000	50.0000	3.8400	0.7110	0.2290	50.0000
BZ	VRC34.01-HC-F25G	VRC34.01-YD13		29.4000	0.1560	50.0000	50.0000	0.8290	0.4350	0.1730	50.0000
BZ	VRC34.01-HC-F25L	VRC34.01-YD14		50.0000	0.2000	50.0000	50.0000	1.1200	0.4950	0.1460	50.0000
BZ	VRC34.01-HC-A40T	VRC34.01-YD15		0.9390	0.3580	50.0000	50.0000	0.3650	0.4570	0.2070	50.0000
BZ	VRC34.01-HC-P41Y	VRC34.01-YD16		1.6900	0.3050	50.0000	50.0000	0.5010	0.3710	0.2220	50.0000
BZ	VRC34.01-HC-T58Q	VRC34.01-YD17		3.9300	0.6170	50.0000	50.0000	0.8500	0.3660	0.2550	50.0000
BZ	VRC34.01-HC-T58V	VRC34.01-YD18		12.8000	0.3540	50.0000	50.0000	0.6140	0.3070	0.1330	50.0000
BZ	**VRC34.01-HC-T59F**	VRC34.01-YD19		0.3710	0.0990	50.0000	50.0000	0.1760	0.1870	0.0850	50.0000
BZ	**VRC34.01-HC-T59Y**	VRC34.01-YD20		0.3550	0.1550	50.0000	50.0000	0.1860	0.1890	0.0900	50.0000
BZ	VRC34.01-HC-T59W	VRC34.01-YD21		0.9520	0.1420	50.0000	50.0000	0.3410	0.3440	0.1640	50.0000
BZ	VRC34.01-HC-T59V	VRC34.01-YD22		0.6650	0.1230	50.0000	50.0000	0.3720	0.3820	0.0870	50.0000
BZ	VRC34.01-HC-T60L	VRC34.01-YD23		50.0000	0.2030	50.0000	50.0000	0.5270	0.3350	0.1300	50.0000
BZ	VRC34.01-HC-T60V	VRC34.01-YD24		1.8800	0.3750	50.0000	50.0000	0.5890	0.2950	0.1170	50.0000
BZ	VRC34.01-HC-S88G	VRC34.01-YD25		0.9610	0.1100	50.0000	50.0000	0.2690	0.2310	0.1360	50.0000
BZ	VRC34.01-HC-T119M	VRC34.01-YD26		1.6600	0.2440	50.0000	50.0000	0.4760	0.3930	0.1340	50.0000
BZ	VRC34.01-LC-D1G	VRC34.01-YD27		1.2200	0.2190	50.0000	50.0000	0.3730	0.3710	0.1690	50.0000
BZ	VRC34.01-LC-D1I	VRC34.01-YD28		1.0200	0.1590	50.0000	50.0000	0.3240	0.2980	0.1070	50.0000
BZ	VRC34.01-LC-D1Y	VRC34.01-YD29		1.1100	0.2140	50.0000	50.0000	0.3830	0.3140	0.1340	50.0000
BZ	VRC34.01-LC-S9D	VRC34.01-YD30		1.2100	0.2740	50.0000	50.0000	0.3390	0.2910	0.1350	50.0000
BZ	VRC34.01-LC-F10I	VRC34.01-YD31		1.0700	0.2440	50.0000	50.0000	0.3630	0.3280	0.1500	50.0000
BZ	VRC34.01-LC-R24D	VRC34.01-YD32		2.7000	0.3270	50.0000	50.0000	0.6420	0.4670	0.2020	50.0000
BZ	VRC34.01-LC-G28H	VRC34.01-YD33		1.3500	0.2210	50.0000	50.0000	0.4050	0.3110	0.1630	50.0000
BZ	VRC34.01-LC-R30A	VRC34.01-YD34		2.3100	0.1820	50.0000	50.0000	0.3160	0.2660	0.1200	50.0000
BZ	VRC34.01-LC-R30F	VRC34.01-YD35		1.3100	0.2130	50.0000	50.0000	0.2900	0.3070	0.1530	50.0000
BZ	VRC34.01-LC-R30Q	VRC34.01-YD36		4.6700	0.4810	50.0000	50.0000	0.4570	0.4540	0.1720	50.0000
BZ	VRC34.01-LC-R30S	VRC34.01-YD37		1.6200	0.2210	50.0000	50.0000	0.4590	0.3690	0.1560	50.0000
BZ	VRC34.01-LC-R30W	VRC34.01-YD38		1.2300	0.2200	50.0000	50.0000	0.4020	0.2730	0.1690	50.0000
BZ	VRC34.01-LC-R30Y	VRC34.01-YD39		1.1800	0.2940	50.0000	50.0000	0.4040	0.2910	0.1090	50.0000
BZ	VRC34.01-LC-K42R	VRC34.01-YD40		1.2400	0.2500	50.0000	50.0000	0.2830	0.3270	0.1840	50.0000
BZ	VRC34.01-LC-N45T	VRC34.01-YD41		0.9580	0.2740	50.0000	50.0000	0.4610	0.4530	0.1580	50.0000
BZ	VRC34.01-LC-L54R	VRC34.01-YD42		0.4740	0.1050	50.0000	50.0000	0.4680	0.3300	0.0970	50.0000
BZ	VRC34.01-LC-S56H	VRC34.01-YD43		0.9700	0.1770	50.0000	50.0000	0.4180	0.2680	0.1240	50.0000
BZ	VRC34.01-LC-T65R	VRC34.01-YD44		0.8190	0.1970	50.0000	50.0000	0.3780	0.2530	0.0910	50.0000
BZ	VRC34.01-LC-T65S	VRC34.01-YD45		1.2700	0.3250	50.0000	50.0000	0.3100	0.3180	0.1320	50.0000
BZ	VRC34.01-LC-S92F	VRC34.01-YD46		50.0000	0.2820	50.0000	50.0000	0.5510	0.3280	0.1750	50.0000
BZ	VRC34.01-LC-S92T	VRC34.01-YD47		0.7170	0.2000	50.0000	50.0000	0.4410	0.3630	0.1460	50.0000
BZ		VRC34.01 wt- BZ		0.6290	0.1010	50.0000	50.0000	0.4320	0.2840	0.1100	50.0000
JM		N123-VRC34.01		0.8960	0.1090	50.0000	50.0000	0.4400	0.3000	0.1070	50.0000

IC80

			clade	A	A	AE	B	C	C	C	D
source	Descriptive ID	Antibody	virus	BB539.2B13.SG3	BG505.W6M.C2.SG3	CNE56.SG3	QH0515.01.SG3	CAP210.E8.SG3	CNE30.SG3	CNE31.SG3	6405.v4.c34.SG3
BZ	VRC34.01-HC-E2K	VRC34.01-YD01		1.1700	0.3770	50.0000	50.0000	0.7120	0.7260	0.4030	50.0000
BZ	**VRC34.01-HC-E2P**	VRC34.01-YD02		1.1500	0.3730	50.0000	50.0000	0.8560	0.7010	0.3250	50.0000
BZ	VRC34.01-HC-V3W	VRC34.01-YD03		50.0000	0.9940	50.0000	50.0000	50.0000	2.0100	1.1400	50.0000
BZ	VRC34.01-HC-V3F	VRC34.01-YD04		50.0000	1.2000	50.0000	50.0000	50.0000	1.7300	1.1600	50.0000
BZ	VRC34.01-HC-G8D	VRC34.01-YD05		50.0000	0.9460	50.0000	50.0000	2.5300	1.1800	0.5810	50.0000
BZ	VRC34.01-HC-K12A	VRC34.01-YD06		50.0000	0.7820	50.0000	50.0000	2.1500	1.5500	0.6240	50.0000
BZ	VRC34.01-HC-K12T	VRC34.01-YD07		50.0000	0.8880	50.0000	50.0000	2.6300	1.4800	0.6500	50.0000
BZ	VRC34.01-HC-K13L	VRC34.01-YD08		50.0000	0.8170	50.0000	50.0000	2.6900	1.4200	0.5770	50.0000
BZ	VRC34.01-HC-P14Q	VRC34.01-YD09		50.0000	0.7840	50.0000	50.0000	1.9500	1.4000	0.5350	50.0000
BZ	VRC34.01-HC-G15E	VRC34.01-YD10		50.0000	0.5180	50.0000	50.0000	2.1800	1.4200	0.4960	50.0000
BZ	VRC34.01-HC-A16D	VRC34.01-YD11		50.0000	1.5300	50.0000	50.0000	3.2900	1.7100	0.5750	50.0000
BZ	VRC34.01-HC-F25A	VRC34.01-YD12		50.0000	0.8710	50.0000	50.0000	50.0000	2.1700	1.4500	50.0000
BZ	VRC34.01-HC-F25G	VRC34.01-YD13		50.0000	1.2000	50.0000	50.0000	9.1800	1.9600	0.7450	50.0000
BZ	VRC34.01-HC-F25L	VRC34.01-YD14		50.0000	1.1200	50.0000	50.0000	42.5000	1.8300	0.7990	50.0000
BZ	VRC34.01-HC-A40T	VRC34.01-YD15		43.8000	0.8020	50.0000	50.0000	2.3200	1.4800	0.5750	50.0000
BZ	VRC34.01-HC-P41Y	VRC34.01-YD16		50.0000	1.2700	50.0000	50.0000	2.5500	1.4600	0.6920	50.0000
BZ	VRC34.01-HC-T58Q	VRC34.01-YD17		50.0000	50.0000	50.0000	50.0000	5.2800	1.2900	0.6710	50.0000
BZ	VRC34.01-HC-T58V	VRC34.01-YD18		50.0000	50.0000	50.0000	50.0000	7.0400	1.1700	0.7340	50.0000
BZ	**VRC34.01-HC-T59F**	VRC34.01-YD19		2.0900	0.4450	50.0000	50.0000	0.9000	0.8280	0.3500	50.0000
BZ	**VRC34.01-HC-T59Y**	VRC34.01-YD20		1.7200	0.5270	50.0000	50.0000	0.8260	0.7670	0.3460	50.0000
BZ	VRC34.01-HC-T59W	VRC34.01-YD21		4.3100	0.8210	50.0000	50.0000	1.9600	1.4600	0.5980	50.0000
BZ	VRC34.01-HC-T59V	VRC34.01-YD22		3.4300	0.4990	50.0000	50.0000	1.4400	1.2400	0.4220	50.0000
BZ	VRC34.01-HC-T60L	VRC34.01-YD23		50.0000	50.0000	50.0000	50.0000	5.7000	1.2700	0.5010	50.0000
BZ	VRC34.01-HC-T60V	VRC34.01-YD24		50.0000	1.9300	50.0000	50.0000	3.1900	1.1300	0.4420	50.0000
BZ	VRC34.01-HC-S88G	VRC34.01-YD25		50.0000	1.0800	50.0000	50.0000	1.6600	0.9440	0.4720	50.0000
BZ	VRC34.01-HC-T119M	VRC34.01-YD26		50.0000	0.5060	50.0000	50.0000	2.4900	1.2700	0.5780	50.0000
BZ	VRC34.01-LC-D1G	VRC34.01-YD27		50.0000	0.7330	50.0000	50.0000	2.1000	1.2300	0.6040	50.0000
BZ	VRC34.01-LC-D1I	VRC34.01-YD28		50.0000	0.7210	50.0000	50.0000	1.7200	0.9890	0.4150	50.0000
BZ	VRC34.01-LC-D1Y	VRC34.01-YD29		50.0000	0.9040	50.0000	50.0000	1.7400	1.1200	0.4720	50.0000
BZ	VRC34.01-LC-S9D	VRC34.01-YD30		50.0000	1.5800	50.0000	50.0000	1.5700	1.0000	0.4430	50.0000
BZ	VRC34.01-LC-F10I	VRC34.01-YD31		50.0000	0.4750	50.0000	50.0000	2.5600	1.3200	0.6210	50.0000
BZ	VRC34.01-LC-R24D	VRC34.01-YD32		50.0000	1.1000	50.0000	50.0000	4.3400	1.8400	0.7590	50.0000
BZ	VRC34.01-LC-G28H	VRC34.01-YD33		50.0000	0.6530	50.0000	50.0000	2.1800	1.0400	0.5730	50.0000
BZ	VRC34.01-LC-R30A	VRC34.01-YD34		50.0000	0.6850	50.0000	50.0000	1.7800	1.0500	0.4200	50.0000
BZ	VRC34.01-LC-R30F	VRC34.01-YD35		50.0000	0.7560	50.0000	50.0000	1.9600	1.1100	0.5190	50.0000
BZ	VRC34.01-LC-R30Q	VRC34.01-YD36		50.0000	1.9200	50.0000	50.0000	3.8500	1.4900	0.5640	50.0000
BZ	VRC34.01-LC-R30S	VRC34.01-YD37		50.0000	1.0900	50.0000	50.0000	2.3600	1.2900	0.5210	50.0000
BZ	VRC34.01-LC-R30W	VRC34.01-YD38		50.0000	1.2200	50.0000	50.0000	1.9600	1.0600	0.4650	50.0000
BZ	VRC34.01-LC-R30Y	VRC34.01-YD39		50.0000	0.6710	50.0000	50.0000	1.8800	1.0700	0.4180	50.0000
BZ	VRC34.01-LC-K42R	VRC34.01-YD40		50.0000	0.9510	50.0000	50.0000	1.7600	1.0100	0.4550	50.0000
BZ	VRC34.01-LC-N45T	VRC34.01-YD41		50.0000	1.7500	50.0000	50.0000	2.2200	1.5100	0.5530	50.0000
BZ	VRC34.01-LC-L54R	VRC34.01-YD42		10.5000	0.6770	50.0000	50.0000	1.6900	1.0700	0.4020	50.0000
BZ	VRC34.01-LC-S56H	VRC34.01-YD43		50.0000	1.1000	50.0000	50.0000	2.3200	1.2200	0.5320	50.0000
BZ	VRC34.01-LC-T65R	VRC34.01-YD44		27.5000	0.5360	50.0000	50.0000	1.7600	0.9550	0.3850	50.0000
BZ	VRC34.01-LC-T65S	VRC34.01-YD45		50.0000	0.9800	50.0000	50.0000	2.1300	1.0600	0.5010	50.0000
BZ	VRC34.01-LC-S92F	VRC34.01-YD46		50.0000	50.0000	50.0000	50.0000	4.5600	1.1100	0.4730	50.0000
BZ	VRC34.01-LC-S92T	VRC34.01-YD47		4.3700	0.7900	50.0000	50.0000	1.7500	1.1400	0.4370	50.0000
BZ		VRC34.01 wt- BZ		50.0000	0.5860	50.0000	50.0000	2.0200	0.9640	0.4570	50.0000
JM		N123-VRC34.01		50.0000	0.6690	50.0000	50.0000	2.2400	1.2700	0.5160	50.0000

Color shading represents potency as follows:

- <0.001
- .001-.01
- .01-.100
- .100-1.00
- 1.00-10.0
- >10.0

Supplementary Table 5. 20-virus predictive neutralization panel design related to Figures 2C, 4C, and S4B.

				Control Antibodies IC50 (ug/mL)								
**Strains**	**Clade**	**FP seq**		**ACS202**	**N123-VRC34.01**	**PGT151**	**DF1W314**	**0PV12**	**0PV20**	**0PV21**	**110D12**	**2712-vFP16.02**
BG505.W6M.C2	A	AVGIGAVF	**FP_v1**	50	0.211	0.004	0.257	15.2	6.07	20.7	2.21	2.51
CNE30	C	AVGLGAVF	other	50	0.191	50	500	500	500	500	5.29	100
BB539.2B13	A	AVGIGAVF	**FP_v1**	50	1.82	0.005	0.566	460	500	122	10.9	100
CAP210.E8	C	AVGIGAVF	**FP_v1**	1.02	0.218	0.032	500	500	101	500	2.66	43.8
CNE31	C	AVGIGAVF	**FP_v1**	0.037	0.122	0.01	314	500	500	500	5.2	100
CNE56	AE	AVGIGAMI	**FP_Thai**	50	500	50	500	3.19	24.6	133	0.65	0.405
6405.v4.c34	D	AIGLGAMF	**FP_v3**	50	500	0.036	28.9	500	500	500	50	100
QH0515.01	B	AVGTIGAMF	**FP_v4**	0.422	500	0.009	500	500	500	500	50	100
C1080.c3	AE	AVGIGAMI	**FP_Thai**	50	5.2	50	70.2	246	321	66.1	50	19
C2101.c1	AE	AVGIGAMI	**FP_Thai**	50	13.8	50	500	500	500	500	50	11.7
C4118.09	AE	AVGIGAMI	**FP_Thai**	0.789	103	50	12	500	500	500	17.1	57.9
A03349M1	D	AIGLGAMF	**FP_v3**	0.147	500	50	13.7	500	500	500	50	100
CNE7	BC	AVGTIGAMF	**FP_v4**	0.179	500	0.021	500	500	500	500	50	100
T266-60	AG	AVGLGAVF	other	50	0.301	0.013	500	500	500	500	1.86	100
DU156.12	C	AVGLGAVL	other	50	500	0.007	500	500	500	500	0.79	78.2
3415.v1.c1	A	AIGMGAVF	other	50	0.126	0.005	500	500	500	500	4.44	100
CAP45.G3	C	AVGIGAVL	other	50	0.064	0.02	500	500	357	500	11.7	6.65
BI369.9A	A	AVGIGAVF	**FP_v1**	50	0.108	0.059	0.118	3.43	1.59	9.59	0.39	0.4
269-12	AG	AIGMGAVF	other	50	72	0.007	500	500	500	500	2.79	100
0077_V1.C16	C	AVGIGAMF	other	50	1.12	0.005	1.64	14.9	1.7	500	0.849	0.643
			20-virus IC50 geomean:	10.36	6.08	0.14	64.26	204.96	172.90	275.83	6.56	24.79
			20-virus breadth	30%	60%	70%	35%	20%	20%	10%	70%	40%

Supplementary Table 6. Crystal structure data collection statistics related to Figure 3 & 6.

	**VCR34.01_mm28 + FP**	**VRC34.01-combo.1 + FP**
**PDB ID**	8F7Z	8ELI
**Data collection**		
Space group	$P2_12_12_1$	$P\ 2_12_12_1$
Cell constants		
a, b, c (Å)	129.9, 130.6, 130.6	38.3, 86.2, 128.8
α, β , γ (°)	90, 90, 90	90, 90, 90
Resolution (Å)	50 – 2.70 (2.80 – 2.70)	50 - 1.49
Rmerge (%)	13.4 (56.1)	0.067 (0.310)
I / σI	16.0 (2.68)	42.8 (5.6)
Completeness (%)	97.85 (96.72)	98.8 (95.1)
Redundancy	6.5 (6.8)	10.7 (10.2)
**Refinement**		
Resolution (Å)	36.22 – 2.70	36.7-1.22
No. reflections	60,161 (5,863)	69,890 (6,940)
R_{work} / R_{free} (%)	21.09 / 24.30	0.16 / 0.18
No. atoms		
Protein	12,968	3,387
Ligand/ion		
Water	346	626
B-factors		
Protein	40.99	15.53
Water	36.88	30.67
R.m.s. deviations		
Bond lengths (Å)	0.006	0.005
Bond angles (°)	0.98	0.830

*Values in parentheses are for the highest-resolution shell

Supplementary Table 7. Cryo-EM data related to Figures 3 & 6.

Cryo-EM data collection, refinement, and validation statistics

	BG505-DS-SOSIP – VRC34.01 (EMD-28617) (PDB 8EUU)	BG505-DS-SOSIP – VRC34.01_combo1 (EMD-28618) (PDB 8EUV)	BG505-DS-SOSIP – VRC34.01_mm28 (EMD-28619) (PDB 8EUW)
**Data collection and processing**			
Magnification	105,000	105,000	105,000
Voltage (kV)	300	300	300
Electron exposure (e–/Å^2)	43.5	43.5	43.5
Defocus range (μm)	-0.7 to -2.0	-0.7 to -2.0	-0.7 to -2.0
Pixel size (Å)	0.415 / 0.83	0.415 / 0.83	0.415 / 0.83
Symmetry imposed	C3	C3	C3
Initial particle images (no.)	1,363,410	3,865,478	3,780,744
Final particle images (no.)	548,906	979,968	918,637
Map resolution (Å)	2.7	2.6	2.7
FSC threshold	0.143	0.143	0.143
**Refinement**			
Initial model used (PDB code)		6V0R	
Model resolution (Å)	2.9	2.8	2.8
FSC threshold	0.143	0.143	0.143
Model composition			
Non-hydrogen atoms	19,935	20,147	20,049
Protein residues	2,400	2,400	2,400
Ligands	87	87	81
B factors (Å^2)			
Protein	26.2	52.1	25.2
Ligand	34.9	49.0	31.7
R.m.s. deviations			
Bond lengths (Å)	0.003	0.006	0.005
Bond angles (°)	0.59	0.77	0.74
Validation			
MolProbity score	1.1	1.2	1.1
Clashscore	2.8	3.0	3.1
Poor rotamers (%)	0.4	0.6	0.4
Ramachandran plot			
Favored (%)	97.8	97.3	98.3
Allowed (%)	2.2	2.7	1.7
Disallowed (%)	0.0	0.0	0.0

Supplementary Table 8. Isothermal Titration Calorimetry (ITC) data, related to Figure 7C.

mAb	*Peptide*	K_d *(nM)*	*ΔG (kcal/mol)*	*ΔH (kcal/mol)*	*-TΔS (kcal/mol)*	*N*
VRC34.01	FP_v1	33	-10.2	-19.1	+8.9	1.0
	FP_v2	37	-10.1	-19.4	+13.3	0.7
	FP_v3F	310	-8.9	-17.2	+8.3	0.9
	FP_v4	137	-9.4	-22.2	+12.8	*
	FP_v3	2,500	-7.6	-12.9	+5.3	1[&]
	FP_Thai	580	-8.5	-15.3	+6.8	0.8
T59F	FP_v1	7.7	-11.1	-23.8	+12.7	0.8
	FP_v2	3.0	-11.6	-24.9	+13.3	0.7
	FP_v3F	31	-10.2	-19.3	+9.1	1.0
	FP_v4	22	-10.4	-23.4	+13.0	*
	FP_v3	540	-8.6	-23.4	+14.8	0.8
	FP_Thai	88	-9.6	-23.3	+13.7	0.9
E2K	FP_v1	28	-10.3	-18.8	+8.5	1.0
	FP_v2	36	-10.2	-21.7	+11.5	0.9
	FP_v3F	650	-8.4	-20.1	+11.7	1.1
	FP_v4	92	-9.6	-21.8	+12.2	*
	FP_v3	1,400	-8.0	-7.0	-1.0	1[&]
	FP_Thai	515	-8.6	-12.2	+3.6	1.0
Combo1	FP_v1	5.1	-11.3	-24.7	+13.4	1.0
	FP_v2	3.0	-11.6	-28.1	+16.5	0.7
	FP_v3F	25	-10.4	-22.1	+11.7	1.0
	FP_v4	21	-10.5	-23.0	+12.5	*
	FP_v3	220	-9.1	-17.2	+8.1	1.0
	FP_Thai	85	-9.6	-21.9	+12.3	1.0
mm28	FP_v1	2.3	-11.8	-30.5	+18.7	0.8
	FP_v2	0.9	-12.3	-31.8	+19.5	1.0
	FP_v3F	6.8	-11.1	-27.5	+16.4	1.0
	FP_v4	18	-10.6	-17.7	+7.1	*
	FP_v3	45	-10.0	-24.7	+14.7	1.0
	FP_Thai	5.8	-11.2	-25.8	+14.6	0.8

*For FP_V4, N is a measure of fraction peptide able to bind (~2%)
[&]N set to 1 during fitting due to weak binding

v1 - V1: AVGIGAVFLGGGKKKGGHHHHHHHH;
v2 - V2: AVGLGAVFLGGGKKKGGHHHHHHHH;
v3F - V3: AIGLGAVFLGGGKKKGGHHHHHHHH;
v4 - V4: AVGTIGAMFLGGGKKKGGHHHHHHHH;
v3 - V3M: AIGLGAMFLGGGKKKGGHHHHHHHH;
Thai - V5Thai: AVGIGAMILGGGKKKGGHHHHHHHH.

Supplemental Table 9. VRC34.01_mm28 resistant strain FP sequence analysis. A Fisher's exact test was performed to examine the significance of association of amino acid mutations with resistance at each FP amino acid residue, for each of the 11 residues of the FP sequence below. A data table was generated of mutations for each position containing the number sensitive and resistant strains for all amino acids observed at that position, and a two-sided Fisher's exact test was then performed using the table for each residue. A Bonferroni adjustment was made for multiple comparisons, with adjusted $p<0.05/11=0.0045$.

Number of sensitive and resistant FP sequences to VRC34.01_mm28

FP seq	# sensitive strains	# resistant strains
A-VG-MAA-LF	1	0
A-A--IGA-LF	0	1
A-A--LGA-LF	0	2
A-A--LGA-VF	0	4
A-AG-IGA-VI	0	1
A-IG-IGA-VF	1	0
A-IG-LGA-LF	1	3
A-IG-LGA-MF	6	1
A-IG-LGA-VF	3	0
A-IG-LGT-VL	1	0
A-IG-MGA-LF	0	1
A-IG-MGA-VF	2	0
A-IG-MGA-VL	1	0
A-MG-IGA-MF	1	0
A-VG-FGA-FF	0	2
A-VG-IGA-LF	6	1
A-VG-IGA-LL	1	0
A-VG-IGA-MF	7	0
A-VG-IGA-MI	15	3
A-VG-IGA-VF	58	0
A-VG-IGA-VI	1	1
A-VG-IGA-VL	4	1
A-VG-IGI-MI	1	0
A-VG-IGT-MI	2	0
A-VG-LGA-FF	0	1
A-VG-LGA-FL	1	0
A-VG-LGA-LF	2	0
A-VG-LGA-MF	3	0
A-VG-LGA-MI	0	1
A-VG-LGA-VF	24	0
A-VG-LGA-VI	2	0
A-VG-LGA-VL	4	0
A-VG-MAA-VF	0	1
A-VG-MGA-LF	1	0
A-VG-MGA-LI	0	1
A-VG-MGA-VF	3	0
A-VG-MGA-VL	1	1
A-VG-VGAVMF	0	1
A-VGGFGA-MI	0	1
A-VGGIGA-VF	1	0
A-VGIVGA-MF	1	0
A-VGMLGA-MF	0	1
A-VGTIGA-MF	2	9
A-VT-IGA-MI	1	0
A-VT-LGA-MF	1	0
A-VT-LGA-VF	1	0
A-VT-MGA-VF	1	1
A-VVELGA-VF	1	0
AVVG-LGA-VF	1	2
D-LG-LGA-LF	0	1
E-VT-LGA-LF	0	1
T-VG-IGA-LF	1	0
T-VG-IGA-VF	1	0

Analysis of resistant amino acids at different positions

Position	Amino acid	# sensitive strains	# resistant strains	Fisher's Test p-values
1st	A	163	41	0.005
	D	0	1	
	E	0	1	
	T	2	0	
1a	-	164	41	0.11
	V	1	2	
2nd	V	149	29	$2*10^{-9}$
	A*	0	8	
	I	15	5	
	M	1	0	
	L	0	1	
3rd	T	4	2	10^{-7}
	V	2	2	
	G	159	32	
	-	0	7	
3a	-	160	32	$3*10^{-8}$
	E	1	0	
	G	1	1	
	I	1	0	
	M	0	1	
	T	2	9	
4th	F	0	3	0.0006
	I	103	17	
	L	51	17	
	M	10	5	
	V	1	1	
5th	A	1	1	0.37
	G	164	42	
6th	A	161	43	1.0
	I	1	0	
	T	3	0	
6a	-	165	42	0.049
	V	0	1	
7th	F	1	3	0.007
	L	13	11	
	M	40	17	
	V	111	12	
8th	F	130	33	0.6
	I	22	8	
	L	13	2	

*Residues in red are associated with resistance to VRC34.01_mm28
