# Supplementary material for: Antibody-directed evolution reveals a mechanism for enhanced neutralization at the HIV-1 fusion peptide site
Source: Nat Commun. 2023 Nov 21;14:7593. doi: 10.1038/s41467-023-42098-5 (PMC10663459; doi:10.1038/s41467-023-42098-5)
Supplement: Supplementary file 3 — Description of Additional Supplementary Files [file 41467_2023_42098_MOESM3_ESM.pdf]

Description of Additional Supplementary Files

File Name: Supplementary Data 1
Description: 20 virus neutralization panel comparisons related to Figures 2C, 4C, and S4B.

File Name: Supplementary Data 2
Description: 208 virus neutralization data for VRC34.01 and VRC34.01_Combo1, related to Figure 2D.

File Name: Supplementary Data 3
Description: 208 virus neutralization data for VRC34.01_mm28, related to Figure 5.
